# Chronic Kidney Disease of Unknown Etiology in a Tertiary Care Teaching Hospital

**DOI:** 10.7759/cureus.35446

**Published:** 2023-02-25

**Authors:** Mohammed A Mughni, Mohammed A Mateen, Mohammed Asifuddin, Khaja K Khan, Ariyan Khan, Maria Khan, Priyadarshi Prajjwal, Raunak Ranjan

**Affiliations:** 1 Internal Medicine, Shadan Institute of Medical Sciences, Teaching Hospital and Research Center, Hyderabad, IND; 2 Internal Medicine, Deccan College of Medical Sciences and Research Center, Hyderabad, IND; 3 Internal Medicine, Bharati Vidyapeeth University Medical College, Pune, IND

**Keywords:** kidney function, chronic kidney disease of unknown aetiology, ngal, kim-1, novel biomarkers

## Abstract

Background

Several primary studies have looked at the burden of chronic kidney disease among diabetic patients, but their results have shown significant variance in India. In order to determine the combined prevalence of chronic kidney disease and associated risk factors among patients with diabetes, this study used a combination of methods.

Methods

Over the course of two years, a cross-sectional observational study was undertaken in the Tertiary Care Teaching Hospital's Department of General Medicine including all chronic kidney disease patients of 18 years of age and above of either gender. People not suffering from the disease were chosen as controls. Kidney Injury Molecule-1 (KIM-1) and neutrophil gelatinase-associated lipocalin-ELISA (NGAL-ELISA) sample analysis by the kit method was done. The study was carried out in accordance with Schedule Y, ICH GCP principles, and the Helsinki Declaration after receiving approval from the institutional ethics committee.

Results

In our study, the urinary mean KIM-1 was 49.75±4.35 μg/g Cr in the Chronic Kidney Disease of Unknown etiology (CKDu) group and 1.43±0.15 μg/g Cr in the controls group. The mean NGAL levels of the CKDu Group and the controls group were 8.94±1.31 μg/g and 0.41±0.05 μg/g, respectively. In CKDu and the controls group, the mean eGFR (ml/min/1.73m^2^) was 69.83±7.91 and 108±3.7, respectively. The mean serum creatinine (mg/dL) was reported 3.79 in the CKDu group and 1.0 in the controls group.

Conclusion

Despite the urban centers previously being thought of as a non-endemic location, for the first time in the city, 60 CKDu patients are reported in this study. This is the first study to use the urinary biomarkers KIM-1 and NGAL to find suspected cases of CKDu and early kidney damage in local communities in the urban centers.

## Introduction

A decreased glomerular filtration rate (GFR) of 60 ml/min/1.73 m^2^ for three months is stated to be chronic kidney disease (CKD), which is characterized as functional or structural anomalies of the kidney. It is a major health issue in global public health [1]. Globally, there were 697.5 million instances of all-stage CKD in 2017, and 1.2 million people per year expired as a result of expensive medical care [2]. Furthermore, it is predicted that being unable to obtain renal replacement therapy will cause between 2.3 and 7.1 million adult deaths before 2030 [3]. Particularly in Latin America, sub-Saharan Africa, and India, the prevalence of CKD has been rising [4].

Studies indicate that a number of factors, including obesity, advanced age, hypertension, diabetes mellitus, male gender, dyslipidemia, HIV infection, usage of nephrotoxic drugs, heavy alcohol use, smoking, family history of kidney disease, electrolyte, and acid-base abnormalities, low-paying employment use of conventional nonallopathic medications, and low hemoglobin, are to blame for CKD, despite the fact that the exact cause of the condition is still unknown [5]. Some of the possible risk factors can easily be identified and treated early, generally at a low cost. Patients with CKD frequently experience reduced quality of life, significantly higher healthcare expenses, and cardiovascular mortality, stroke, ischemic heart disease, gout, depression peripheral vascular disease, and anxiety are all at a higher risk [6].

The proximal tubule apical membrane expresses kidney injury molecule-1 (KIM-1), a type 1 transmembrane protein, when there is kidney injury [7]. KIM-1 was demonstrated to be an exceptional predictor of histological alterations in serum creatinine and blood urea nitrogen in the proximal tubule in response to various pathophysiological circumstances or toxicants [8].

The 25 kDa protein known as neutrophil gelatinase-associated lipocalin (NGAL) is a member of the lipocalin superfamily. Although many other cells, such as renal tubular cells, can create NGAL in response to numerous stresses, it was initially discovered in active neutrophils. Additionally, it has been found to be involved in kidney development and damaged tubular regeneration. Later clinical studies found urine NGAL to be an early marker of acute kidney injury (AKI). In light of recent data, it may also serve as an investigation for a number of various renal and non-renal disorders [9].

## Materials and methods

It is a prospective observational study done in the Department of General Medicine at Tertiary Care Teaching Hospital over a period of two years.

Inclusion and exclusion criteria

The inclusion and exclusion criteria are summarized in Table 1.

**Table 1:** Inclusion and exclusion criteria

Inclusion Criteria	Exclusion Criteria
All chronic kidney disease patients above 18 years of age of either gender	Known hypertensive patients
	Nephrotic or nephritic syndrome patients
	Kidney damage caused by a snake bite or other nephrogenic toxins.
	Urological disease of known etiology

Methodology

The constituents of the study that are most probable to produce reliable results are organized in a systematic manner by the research methodology. This study provides a concise overview of the materials and procedures used to assess the prevalence of CKD with an unknown etiology among hospitalized patients. Sixty different CKDu patients from the city of Southern Province were used in the case group. The groups were segregated using the random sampling technique and the two groups were based on relevant factors such as age, gender, medical history, and other relevant variables to ensure that the two groups were as similar as possible, except for the intervention being tested. KIM-1 and NGAL-ELISA sample analysis (kit method) was used.

Statistical analysis

The gathered information was organized in a Microsoft Excel 2010 (Microsoft® Corp., Redmond, WA) spreadsheet, and SPSS version 27.0 (IBM Corp., Armonk, NY) was used to analyze it. Frequency and percentage were used to represent the qualitative data. Additionally, it was displayed using typical images like bar diagrams and pie charts.

## Results

In our study, the subjects' demographics are presented in Table 2, the mean age of the subjects, and the percentage of males and females were statistically not significant differences between the CKD and control groups (all P > 0.05). In CKDu out of 60, 36 (60%) males and 24 (40%) females were in the CKDu group, and in the control group, 42 (70%) were males and 18 were females (30%) (Table 2). Most of the patients were 30-40 years old, i.e., 20 out of 60 (33.3%), followed by 41-50 years old, i.e., 15 out of 60 (25%) in the CKDu Group (Table 3). The characteristics of the patients in the CKDu group and the control group are shown in Table 4. The distribution of various CKD stages and the distribution of the urinary albumin/creatinine ratio (ACR) are mentioned in Table 5 and Table 6, respectively. In Table 7, we have summarized data of KIM-1 normalized to serum creatinine, ACR, KIM-1, NGAL, and eGFR. The urinary KIM-1 was 49.75±4.35 μg/g Cr in the CKDu group and 1.43±0.15 μg/g Cr in the controls group. NGAL levels of the CKDu group and controls group were 8.94±1.31 μg/g and 0.41±0.05 μg/g, respectively. eGFR (ml/min/1.73m^2^) in CKDu group and controls group were 69.83±7.91 and 108±3.7, respectively. The mean serum creatinine (mg/dL) in the CKDu group was 3.79 and in the controls group was 1.0.

**Table 2:** Distribution of gender between the CKDu group and control group CKDu: Chronic Kidney Disease of Unknown etiology

Gender	CKDu Group (n=60)	Control (n=60)	p-value
Male	36 (60%)	42 (70%)	0.983
Female	24 (40%)	18 (30%)	
Total	60 (100%)	60 (100%)	

**Table 3:** Distribution of different age groups between the CKDu group and control group CKDu: Chronic Kidney Disease of Unknown etiology

Age	CKDu Group (n=60)	Control (n=60)	p-value
30-40 years	20 (33.3%)	14 (23.3%)	0.864
41-50 years	15 (25.0%)	16 (26.6%)	0.789
51-60 years	17 (28.3%)	12 (20.0%)	0.975
>61 years	08 (13.3%)	18 (30.0%)	0.051
Total	60 (100%)	60 (100%)	-

**Table 4:** Characteristics of patients between the CKDu and control group CKDu: Chronic Kidney Disease of Unknown etiology

Characteristics	CKDu Group (n=60)	Control (n=60)
Smoking	18	20
Alcohol Consumption	12	10
Source of drinking water:		
a) Tap	15	13
b) Bore water	15	17

**Table 5:** Distribution of CKD stages CKDu: Chronic Kidney Disease of Unknown etiology

CKD stages	CKDu Group (n=60)
Stage G1 (more than or equal to 90 mL/min/1.73 m^2^)	9
Stage G2 (eGFR 60–89 mL/min/1.73 m^2^)	11
Stage G3a (eGFR 45–59 mL/min/1.73 m^2^)	19
Stage G3b (eGFR 30–44 mL/min/1.73 m^2^)	14
Stages G4 and G5 (eGFR <30 mL/min/1.73 m^2^)	8

**Table 6:** Distribution of urinary albumin/creatinine ratio (ACR) CKDu: Chronic Kidney Disease of Unknown etiology

Urinary ACR	CKDu Group (n=60)	Control (n=60)	P-value
A1 (<30 mg/g)	12 (20%)	48 (80%)	0.493
A2 (30–300 mg/g)	22 (36.6%)	8 (13.3%)	0.592
A3 (>300 mg/g)	26 (43.3%)	4 (6.6%)	0.655
Total	60 (100%)	60 (100%)	0.453

**Table 7:** Clinical information in the controls and CKDu group, as well as creatinine-adjusted tubular indicators (KIM-1 & NGAL) CKDu: Chronic Kidney Disease of Unknown etiology; KIM-1: Kidney Injury Molecule-1; NGAL: Neutrophil gelatinase-associated lipocalin.

Characteristics	CKDu Group (n=60)	Control (n=60)	P-value
KIM-1 (μg/g Cr)	49.75 ± 4.35	1.43 ± 0.15	<0.0001
	7.41–88.66	0.6–8.82	
NGAL (μg/g Cr)	8.94 ± 1.31	0.41 ± 0.05	<0.0001
	0.55–28.44	0.05–0.91	
eGFR (ml/min/1.73m^2^)	69.83 ± 7.91	108 ± 3.7	<0.0001
	60–114	95–139	<0.0001
Serum creatinine (mg/dL) (Mean ± SD)	3.79 ± 0.32	1.0 ± 0.13	<0.0001

## Discussion

The present study helps to shed light on the function of innovative urine biomarkers (NGAL and KIM-1) in the earliest discovery of CKDu in Andhra Pradesh. Additionally, this is the first comparative study in India to examine chronic kidney disease with unknown etiology (CKDu) using a case definition based on WHO criteria.

Males are more susceptible to CKDu in our study than females. In a similar study by Harambat et al. done in NCP, men are more likely to have CKDu (6%) than women (2.9%) [10]. The prevalence of CKDu was higher in males (25.7%) than in females (11.8%) in a study by Mills et al. too [11]. According to a meta-analysis-based inquiry that looked at 68 papers, men with non-diabetic renal illness had much faster kidney function decline over time than women [12]. Males' faster progression from the initial stages of kidney damage to the chronic phases of kidney injury was possibly caused by continual experience due to their rigorous environmental or occupational stressors [13]. As a result, the above-mentioned meta-analysis study excluded women and children and specifically concentrated on male farmers [12]. Our region, which is in the dry zone, uses farming methods that are very similar to those of the CKDu endemic region. As a result, the risk of CKDu appearing in our city is growing.

Based on the WHO's classification of CKDu as well as elevated levels of KIM-1 and NGAL, we present here a study report of 60 different CKDu patients from the city of Southern Province. Diabetes, hypertension, pyelonephritis, renal calculi, etc. are a few examples of co-morbid conditions that may affect urine KIM-1 and NGAL levels [14]. In order to identify and remove instances with co-morbid conditions, we used questionnaires and assessments based on personal medical histories. A recognized initial non-invasive investigation to identify CKD is the measurement of albumin levels [15]. Albuminuria testing as a broad population indicator of renal disease is also supported by an epidemiological investigation [16]. The integrity of the kidneys' proximal tubules and glomerulus is determined by the amount of albumin in the urine [17], which is a gradient that clearly distinguishes the NGAL and KIM-1 values in the cases from the controls. In general, higher urinary KIM-1 levels may signify proximal tubular injury, whereas higher urinary NGAL levels may be caused by observable damage to the distal convoluted tubule and Henle loop.

Drastically increased levels of NGAL and KIM-1 in the urine were also used to confirm instances with CKDu. Both markers made it simple to identify the suspected instances. Farmers in emerging regions of urban centers with normal serum creatinine and eGFR as well as in apparent good health displayed higher levels of NGAL and KIM-1 in comparison to the two control groups, suggesting probable initial kidney injury. It might imply that excessive albumin filtration precedes tubular damage expressed by KIM-1. Similar cases with high urine NGAL, IL-18, and NAG levels have been reported among Nicaraguan sugarcane cutters [18]. Recently, the detection of urinary KIM-1 utilising the micro-urine nanoparticle revealing approach has been reported, however, comparisons cannot be made because of the study's smaller sample size. Due to ischemia injury, KIM-1 is noticeably upregulated in the kidneys [19, 20].

However, only in CKDu with an eight-fold increase over the control group, did NGAL elevation become apparent. Similar findings were found by Bernardi et al., where NGAL levels in CKD sufferers were 26% higher [21]. In another study, sugarcane farmers had 1.49 times greater NGAL, according to Alobaidi et al. [22]. However, no research utilising NGAL in local populations in India has been published. Elevated NGAL levels suggest that damaged tubules will be re-epithelialized, and iron that was lost owing to injury to proximal epithelial tubule cells will be absorbed, and iron-dependent nephrogenesis will be triggered subsequently [23].

Limitations

The absence of established urine biomarker levels that suggest subclinical impairment in nephropathy in the local regions in which our study was carried out was a major study constraint. With the exception of a few recent studies, no prior research has been conducted in the region using comparable occupational cohorts. A follow-up study is necessary because it was also uncertain how each subject's short-term individual variance changed over time. Because only native male farmers in specific farming areas of our city were included in the current study, generalizing the results to the general population and other geographic places may be difficult.

## Conclusions

This is the first study to use the urine biomarkers KIM-1 and NGAL to identify suspected instances of CKDu and to detect early kidney impairment in our local populations using the mentioned markers. According to the findings of our cross-sectional study, high urinary ACR levels were substantially linked with the tubular damage indicated by urinary NGAL and KIM-1 markers. Urinary tubular indicators, found in farming communities in the city, confirm tubulointerstitial illness with tubular injury.
